# NLRP3 Inflammasome Formation and Activation in Nonalcoholic Steatohepatitis: Therapeutic Target for Antimetabolic Syndrome Remedy FTZ

**DOI:** 10.1155/2018/2901871

**Published:** 2018-07-22

**Authors:** Yu Chen, Xingxiang He, Xinxu Yuan, Jinni Hong, Owais Bhat, Guangbi Li, Pin-Lan Li, Jiao Guo

**Affiliations:** ^1^Department of Gastroenterology, The First Affiliated Hospital of Guangdong Pharmaceutical University, Guangzhou, Guangdong 510080, China; ^2^Department of Pharmacology and Toxicology, School of Medicine, Virginia Commonwealth University, Richmond, VA 23298, USA; ^3^Department of Medicine, Guangdong Pharmaceutical University, Guangzhou, Guangdong 510006, China; ^4^Guangdong Metabolic Diseases Research Centre of Integrated Chinese and Western Medicine, Joint Laboratory of Guangdong Province and Hongkong and Macao Regions on Metabolic Diseases, Guangdong Pharmaceutical University, Guangzhou 510006, China

## Abstract

The Nod-like receptor protein 3 (NLRP3) inflammasome activation not only serves as an intracellular machinery triggering inflammation but also produces uncanonical effects beyond inflammation such as changing cell metabolism and increasing cell membrane permeability. The present study was designed to test whether this NLRP3 inflammasome activation contributes to the “two-hit” injury during nonalcoholic steatohepatitis (NASH) and whether it can be a therapeutic target for the action of Fufang Zhenzhu Tiaozhi (FTZ), a widely used herbal remedy for hyperlipidemia and metabolic syndrome in China. We first demonstrated that NLRP3 inflammasome formation and activation as well as lipid deposition occurred in the liver of mice on the high-fat diet (HFD), as shown by increased NLRP3 aggregation, enhanced production of IL-1*β* and high mobility group box 1 (HMGB1), and remarkable lipid deposition in liver cells. FTZ extracts not only significantly reduced the NLRP3 inflammasome formation and activation but also attenuated the liver steatosis and fibrogenic phenotype changed. In *in vitro* studies, palmitic acid (PA) was found to increase colocalization of NLRP3 components and enhanced caspase-1 activity in hepatic stellate cells (HSCs), indicating enhanced formation and activation of NLRP3 inflammasomes by PA. PA also increased lipid deposition. Nlrp3 siRNA can reverse this effect by silencing the NLRP3 inflammasome and both with FTZ. In FTZ-treated cells, not only inflammasome formation and activation was substantially attenuated but also lipid deposition in HSCs was blocked. This inhibition of FTZ on lipid deposition was similar to the effects of glycyrrhizin, an HMGB1 inhibitor. Mechanistically, stimulated membrane raft redox signaling platform formation and increased O_2_^•−^ production by PA to activate NLRP3 inflammasomes in HSCs was blocked by FTZ treatment. It is concluded that FTZ extracts inhibit NASH by its action on both inflammatory response and liver lipid metabolism associated with NLRP3 inflammasome formation and activation.

## 1. Introduction

Nonalcoholic fatty liver disease (NAFLD) is the most common liver disease throughout the world. NAFLD may either be present as a simple steatosis (nonalcoholic fatty liver) or evolves towards its inflammatory complication (10–20%), namely, nonalcoholic steatohepatitis (NASH), which can further progress towards liver cirrhosis and hepatocellular carcinoma, a complication that occurs increasingly in the noncirrhotic NAFLD population [1]. It is generally accepted that the pathogenesis of NASH is involved in a two-step process, which is referred to as a “two-hit” model. The first “hit” is associated with excessive triglyceride or other lipid accumulation in the liver, and the second “hit” leads to the development of liver inflammation and fibrosis, which is attributed to several important pathogenic factors that can eventually induce liver damage such as inflammatory cytokines, oxidative stress, mitochondrial dysfunction, and/or endoplasmic reticulum stress. Recent studies have indicated that the Nod-like receptor protein 3 (NLRP3) inflammasome activation may play a fundamental role in the development of NASH [2, 3]. Since NLRP3 inflammasome has been reported to not only activate the inflammatory response but also possess noncanonical or noninflammatory action that may contribute to the progression of some chronic degenerative or fibrotic diseases [4–7], it is possible that the activation of NLRP3 inflammasome mediates NASH development via the “two-hit” mechanism. We hypothesized that not only hepatitis and consequent fibrosis but also liver steatosis in the progression of NASH may be triggered or modulated by NLRP3 inflammasome activation. In this regard, recent studies indeed demonstrated that in addition to classical inflammatory cytokines such as IL-1*β* and IL-18, HMGB1 released during NLRP3 inflammasome activation is also importantly implicated in both liver steatosis and subsequent hepatitis or fibrosis [8–10]. These inflammatory and uncanonical or noninflammatory effects of NLRP3 inflammasomes on the development of NASH has been the main theme in the present study.

The noncanonical effects during NLRP3 inflammasome activation may answer a long-lasting question of why classic anti-inflammatory medicines, such as commonly used indole and arylpropionic acid derivatives, are not very efficient in the prevention or treatment of many degenerative diseases including NASH, where chronic inflammation are its hallmarks. It may be promising to target the NLRP3 inflammasome and thereby block the “two-hit” mechanisms during NASH. In this regard, a candidate may be Fufang Zhenzhu Tiaozhi (FTZ), a widely used herbal remedy for hyperlipidemia and metabolic syndrome in China, which showed its cocktail therapeutic efficiency. FTZ that has been patented in both the USA and China is a mixture extracted from the Chinese herbal prescription, consisting of *Rhizoma coptidis*, *Fructus Ligustri Lucidi*, *Herba cirsii japonici*, *Radix Salvia miltiorrhiza*, Radix Notoginseng, Cortex Eucommiae, Fructus Citri Sarcodactylis, and *Radix Atractylodes macrocephala*. FTZ has been prescribed over the last 15 years for treatment of hyperlipidemia and metabolic syndrome and related complications such as atherosclerosis and NASH [11, 12]. In a recent study, some components of FTZ were found to prevent the development of fatty liver in rats [13]. However, the mechanism mediating its action remains unknown. In the present study, after characterization of roles in which NLRP3 inflammasomes play in NASH, we also examined whether FTZ prevents NASH development by targeting the effects of NLRP3 inflammasome activation on both inflammatory response and steatosis in the liver.

## 2. Material and Methods

### 2.1. Animals

C57BL/6J mice (8 weeks of age, male or female) were fed a normal diet (ND) or a high-fat diet (HFD, number D12492, Research Diets, NJ, USA) for 4 weeks, and then FTZ extracts (100 mg/kg/day) were fed by gavage for the last 4 weeks both with HFD. The preparation of FTZ extracts for mice was consistent with the protocol described previously [14]. All mice were randomly distributed to different experimental groups. At the endpoint of the experimental period, blood samples were collected and these mice were then sacrificed for harvest of the liver tissues, which were used for oil red O staining, immunofluorescence staining, and biochemical analysis. All protocols were approved by the Institutional Animal Care and Use Committee of the Virginia Commonwealth University.

### 2.2. Cell Culture and Treatments

Mouse hepatic stellate cells (HSCs) were prepared by the discontinuous density gradient centrifugation technique as previously described, and some minor modifications were made to increase success rate as we described previously [4]. The collected cells were cultured in DMEM (Gibco, Carlsbad, CA, USA) containing 10% FBS (Gibco) in humidified 95% air and 5% CO_2_ mixture at 37°C. The cell viability, as measured by a Trypan Blue exclusion assay, was approximately 90%. HSCs were treated with palmitic acid (PA, 200 *μ*M/ml) for indicated hours. HSCs were characterized and confirmed as previously described [15]. We chose to work on HSCs, because they are a major cell type responsible for the progression of liver fibrosis. Our previous studies have shown that NLRP3 inflammasome activation is mainly responsible for the development of liver fibrosis [3, 4]. Our preliminary experiments demonstrated that the optimum response of inflammasome activation occurred over 24-hour PA treatment in HSC cultures, and therefore all experiments in our cell study protocols used the same duration of PA before and after treatments of FTZ extracts (50 *μ*g/ml, prepared by DMSO and diluted with medium in 1 : 1000) or administration of inflammasome inhibitors or blockers such as ROS scavenger N-acetyl-L-cysteine (NAC, 10 *μ*M, Sigma, St. Louis, MO, USA).

### 2.3. Oil Red O Staining

For oil red O staining, the liver tissue slides and HSCs grown on a chamber with coverslips were used as described previously [16] with minor modifications. HSCs (10^4^ cells/well) cultured in a chamber with glass coverslips were treated as indicated and loaded with PA for 24 hours. Frozen liver tissue slides and the HSC coverslips were then stained with oil red O (0.1% in isopropanol) for determination of lipid accumulation. The oil red O staining was examined by light microscopy, and images were obtained by MetaMorph 6.0. The data was represented by the area percentage of each cell positive for oil red O stain, which was calculated in Image Pro Plus 6.0 software (Media Cybernetics, Bethesda, MD, USA). For each sample, at least 200 cells were analyzed and summarized oil red O positive cell counts were used for statistical analysis.

### 2.4. Confocal Microscopic Analysis

For confocal analysis of inflammasome molecule colocalization or aggregation, the liver tissue slides and HSCs grown on a chamber with coverslips were used. They were first fixed in 4% paraformaldehyde in phosphate-buffered saline (PFA/PBS) for 15 min. After being permeabilized with 0.1% Triton X-100/PBS and rinsed with PBS, the slides were incubated overnight at 4°C with anti-NLRP3 (1 : 200, Abcam, MA, USA) and anti-ASC (1 : 50, Enzo, PA, USA) or anti-caspase-1 (1 : 100, Abcam). After washing, these slides incubated with primary antibodies were then incubated with Alexa-488- or Alexa-555-labeled secondary antibodies for 1 h at room temperature. The slides were mounted and subjected to examinations using a confocal laser scanning microscope (Fluoview FV1000, Olympus, Japan) with photos being taken and the colocalization of NLRP3 with ASC or caspase-1 analyzed by the Image-Pro Plus 6.0 software (Media Cybernetics, Bethesda, MD, USA). The summarized data of molecular colocalization efficiency was expressed as correlation coefficient as we described previously [17–19].

*FLICA Staining*. During the last hour of incubation, cells were labeled with FAM-YVAD-fmk caspase-1 FLICA™ kit (Immunochemistry, Bloomington, IN, USA) according to the manufacturer's guidelines, which binds activated caspase-1. Stained cells were visualized by confocal microscopy for active caspase-1 oligomerization, which was colocalized with fibrotic markers vimentin (with antibody staining at 1 : 200) and *α*-smooth muscle actin (*α*-SMA, with antibody at 1 : 200).

*Immunofluorescent Microscopic Analysis of Membrane Raft (MR) Clusters*. HSCs were grown on glass coverslips. After fixation with 4% PFA, cells were incubated with Alexa Fluor 488-conjugated cholera toxin B (Alexa488-CTXB, 2 *μ*g/ml, 2 h, Molecular Probes, CA, USA), which binds with the MR-enriched ganglioside G_M1_. For dual-staining detection of the colocalization of MRs with gp91^phox^ and p47*^phox^*, the cells were first incubated with Alexa488-CTXB and then with anti-gp91^phox^ and p47*^phox^* (1: 200, BD Biosciences, CA, USA), respectively, which was followed by corresponding Alexa555-conjugated secondary antibodies (1: 500, Invitrogen, NY, USA). Then, the colocalization was visualized with confocal microscopy [20, 21].

### 2.5. Immunohistochemistry

Liver tissues were fixed in 4% (*v*/*v*) paraformaldehyde (PFA) in PBS and embedded with paraffin, which were then sliced into tissue sections (4 *μ*M) and mounted on glass slides. These tissue slides were stained with goat anti-IL-1*β* antibody (1 : 100, R&D Systems) overnight at 4°C after a 20 min wash with 3% H_2_O_2_ and 30 min blocking with 10% serum and then probed with anti-goat Ig-G second antibody labeled with HRP according to the protocols described previously [18, 22]. Negative controls were prepared without the primary antibodies. The area percentage of the positive staining was calculated in Image Pro Plus 6.0 software.

### 2.6. Western Blot Analysis

Proteins from cell lysates were denatured with SDS buffer and boiled for 5 minutes. Samples were run on a SDS-PAGE gel, transferred onto polyvinylidene difluoride (PVDF) membrane, and blocked with 5% milk. Then, the membranes were probed with the following primary antibodies overnight at 4°C: rabbit anti-caspase-1 (1 : 500, Santa Cruz, CA, USA) and rabbit anti-*β*-actin (1 : 10000, Santa Cruz). They were incubated with goat anti-rabbit-HRP Ig-G (1 : 5000, Santa Cruz) for 1 hour at room temperature. Immunoreactive bands were detected by chemiluminescence techniques after washing three times and then visualized on Kodak Omat X-ray film. The intensity of the specific bands was calculated using ImageJ software version 1.44p.

### 2.7. Il-1*β* Elisa

After PA treatment with or without pretreatments of inhibitors, the cell supernatant was also collected to measure IL-1*β* production by a mouse IL-1β ELISA kit (Bender MedSystems, CA, USA) according to the protocol described by the manufacturer. The data was expressed as the fold change compared with control cells.

### 2.8. Electromagnetic Spin Resonance (ESR) Spectrometry

For detection of NADPH oxidase-dependent O_2_^•−^ production, protein was gently isolated from 1 × 10^6^ cells and resuspended with modified Krebs–4-(2-hydroxyethyl)-1-piperazineethanesulfonic acid buffer containing deferoximine (100 *μ*M, Sigma) and diethyldithiocarbamate (5 *μ*M; Sigma). A spin trap, 1-hydroxy-3- methoxycarbonyl-2,2,5,5-tetramethylpyrrolidine (CMH, NOXygen, Elzach, Germany) (1 mM final concentration) was then added to the mixture in the presence or absence of manganese-dependent superoxide dismutase (SOD, 200 U/ml; Sigma). The mixtures were loaded into glass capillaries and immediately analyzed for O_2_^•−^ formation kinetics for 10 min in a MiniScope MS200 ESR spectrometer (Magnettech, Berlin, Germany). The ESR spectrometer was set according to the following parameters: biofield, 3350; field sweep, 60 G; microwave frequency, 9.78 GHz; microwave power, 20 mW; modulation amplitude, 3 G; 4096 points of resolution; and receiver gain, 50. The ESR spectrometric signal was recorded in arbitrary units, and the final results were expressed as the fold changes of the treatment group compared to control as we described previously [23].

### 2.9. Statistics

Data are presented as means ± standard error mean. The significant differences between and within multiple groups were examined using one-way or two-way ANOVA, followed by Duncan's multiple-range test. *p* < 0.05 was considered statistically significant.

## 3. Results

### 3.1. NLRP3 Inflammasome Formation and Activation in the Liver of Mice on the HFD with and without Treatment of FTZ

**Figure 1:**
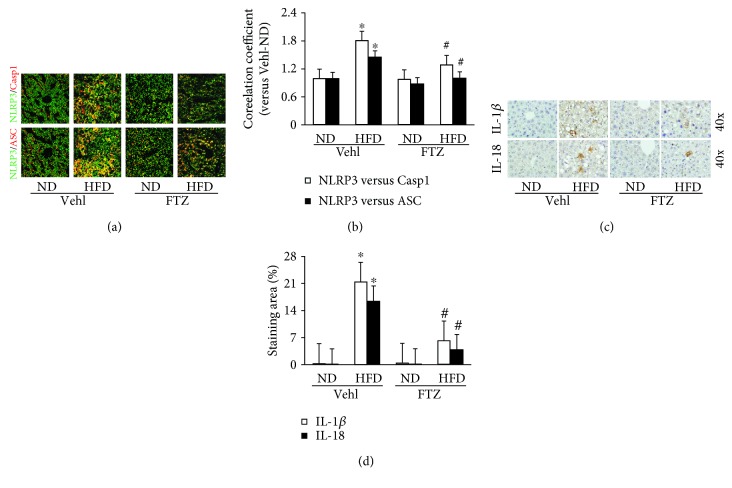
NLRP3 inflammasome formation and activation in the liver from mice on the HFD with and without FTZ treatment. (a) Representative confocal fluorescence images show the colocalization of NLRP3 with caspase-1 or ASC. (b) Correlation coefficient showing a statistically significant increase in NLRP3 inflammasome formation in HFD for 8 weeks with and without FTZ treatment (100 mg/kg/day) by gavage for 4 weeks (*n* = 5 mice per group). (c) Representative immunohistochemical images show positive stain of IL-1*β* and IL-18 in the liver. (d) Positive staining area of IL-1*β* and IL-18 showing a statistically significant increase after NLRP3 inflammasome formation in the liver of mice on the HFD, which was suppressed by FTZ (*n* = 4 mice per group). ^∗^ *p* < 0.05 versus ND-Vehl group; ^#^ *p* < 0.05 versus HFD-Vehl group.

By confocal microscopy, we found that there was significantly elevated colocalization of NLRP3 with ASC or caspase-1 in the liver of mice on the HFD compared with mice on the ND, indicating enhanced formation of NLRP3 inflammasomes. In mice receiving FTZ, HFD-induced increases in colocalization of NLRP3 inflammasome components were substantially blocked (Figure 1(a)). Quantitation of the NLRP3 colocalization by measurement of correlation coefficient is presented in Figure 1(b), showing that NLRP3 inflammasome formation was significantly enhanced in mice on the HFD diet compared to mice on the ND and this enhanced NLRP3 inflammasome formation in the liver of mice on the HFD was significantly attenuated by FTZ.

Immunohistochemical analysis showed that both IL-1*β* and IL-18 (Figure 1(c)) levels significantly increased around fatty hepatocytes in the liver of mice on the HFD, suggesting activation of the inflammasome in these cells. FTZ treatment remarkably reduced this HFD-induced increase in hepatic IL-1*β* and IL-18. As shown in Figure 1(d), the positive staining areas of hepatic IL-1*β* and IL-18 were significantly increased in mice on the HFD without treatment of FTZ. In mice receiving FTZ, the increased IL-1*β* and IL-18 staining in the liver of mice on the HFD was significantly suppressed. It is clear that FTZ can inhibit the activation of NLRP3 inflammasomes in the liver.

### 3.2. Steatosis and NASH in the Liver of Mice on the HFD with and without FTZ Treatment

**Figure 2:**
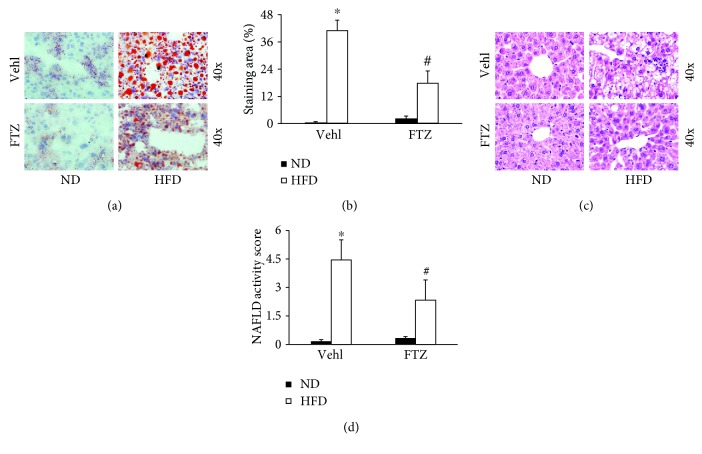
Steatosis and hepatitis in the liver from mice on the HFD with and without FTZ treatment. (a) Representative oil red O staining shows positive staining of lipid deposition in the liver. (b) Positive staining area of lipid deposition showing statistically significant increases after HFD, which was suppressed by FTZ (*n* = 5 mice per group). (c) Representative H&E staining images showing inflammatory infiltration and fatty bulbs in liver cells after HFD which was attenuated by FTZ. (d) NAFLD activity score showing statistically significant increases after HFD for 8 weeks with and without FTZ treatment (*n* = 5 mice per group). ^∗^ *p* < 0.05 versus ND-Vehl group; ^#^ *p* < 0.05 versus HFD-Vehl group

Steatosis as an important pathological change of NASH was analyzed in mice on the HFD. By oil red O staining, it was found that HFD caused a significant increase in lipid deposition in the liver of mice without treatment of FTZ. This enhanced oil red O staining in the liver was markedly reduced when mice were treated with FTZ (Figure 2(a)). The quantitation of tissue areas stained by oil red O showed that more than 40% of the liver in mice on the HFD were with lipid deposition. Treatment of mice with FTZ significantly attenuated this HFD-induced lipid deposition in the liver (Figure 2(b)). It is clear that FTZ significantly prevented steatosis in mice fed with HFD.

By H&E staining, we also examined the morphological changes in the liver of mice from different experimental groups. As shown in Figure 2(c), besides lipid deposition the liver from mice on the HFD exhibited obvious inflammatory cell infiltration and increases in protein leakage into interstitial space. However, the liver from mice receiving FTZ almost lacked these inflammatory changes.  Figure 2(d) depicts the results from the analysis of NAFLD activity score, showing that the increase in NAFLD activity score was highly significant in the liver of mice fed the HFD and that FTZ treatment significantly inhibited such increase in NAFLD activity score.

### 3.3. Fibrotic Changes Associated with NLRP3 Inflammasome Activation in the Liver of Mice on the HFD with and without FTZ Treatment

**Figure 3:**
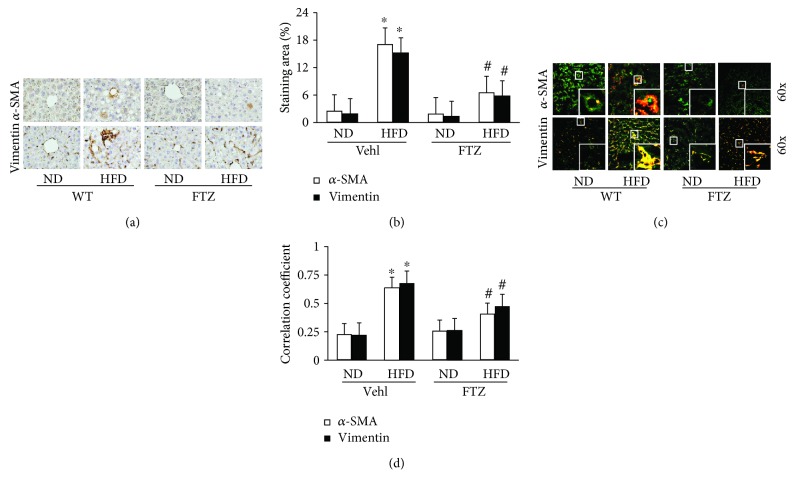
Fibrogenic phenotypes in the liver from mice on the HFD with and without FTZ treatment. (a) Representative immunohistochemical images showing enhanced *α*-SMA and vimentin staining, which are the markers of phenotype changed from quiescent to activated in HSCs. FTZ reduced HFD-induced increase in *α*-SMA and vimentin staining. (b) Summarized data depicting a significant increase in *α*-SMA and vimentin level in the liver of mice on the HFD with and without FTZ treatment (*n* = 4 mice per group). (c, d) Representative images and PPC showing FLICA, active caspase-1 colocalization with α-SMA or vimentin staining in the liver of mice on the HFD, which was reduced by FTZ (*n* = 4 mice per group). ^∗^ *p* < 0.05 versus ND-Vehl group; ^#^ *p* < 0.05 versus HFD-Vehl group.

In addition to steatosis and inflammatory response detected, we also examined whether there is fibrogenic pathology in the liver from different experimental groups of mice. It was found that in the hepatic interstitium, in particular, in tissues around liver sinuses, there were increased levels of *α*-SMA and vimentin, as shown by immunohistochemical staining (Figure 3(a)). It is well known that increased *α*-SMA and vimentin indicate phenotype changes of cells from quiescent to activated status, which occurred more remarkably in HSCs. These results were semiquantitated by measurement of *α*-SMA and vimentin staining areas in the liver. It was shown that *α*-SMA and vimentin levels in the liver were significantly elevated in mice on the HFD, and this HFD-induced fibrotic change in the liver was significantly blocked by FTZ treatment (Figure 3(b)).

Since previous studies have shown that the formation and activation of NLRP3 inflammasomes in fibroblasts including HSCs may trigger the development of liver fibrosis [4], we determined whether these inflammasome-triggered fibrogenetic effects occur in NAFLD. By confocal microscopy, FLICA that indicates activated caspase-1 (green) was found to colocalize with increased *α*-SMA or vimentin (red) around liver sinuses and portal venules of the liver from mice on the HFD as shown by yellow spots (Figure 3(c)). In the liver from mice treated with FTZ, this enhanced colocalization of FLICA with *α*-SMA or vimentin was substantially reduced. By quantitative analysis of this colocalization of FLICA with *α*-SMA or vimentin, we found that the areas or cells with inflammasome activation had much higher level of *α*-SMA or vimentin, indicating the fibrogenesis during this model of NAFLD. FTZ significantly blocked this HFD-induced fibrogenic effect (Figure 3(d)).

### 3.4. PA-Induced NLRP3 Inflammasomes Formation and Increased Caspase-1 Activity in HSCs

**Figure 4:**
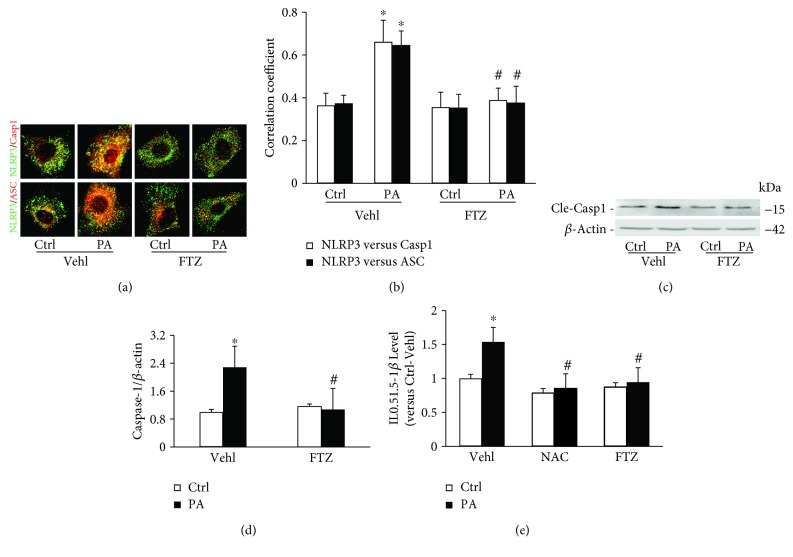
NLRP3 inflammasome formation and activation in HSCs after PA-induced steatosis with and without FTZ treatment. (a) Representative confocal fluorescence images show the colocalization of NLRP3 with caspase-1 or ASC in HSCs. (b) Correlation coefficient (PCC) showing a statistically significant increase in NLRP3 inflammasomes formation in PA-treated (200 mM/ml, 24 hours) HSCs, which was almost completely blocked by FTZ (50 *μ*g/ml, 24 hours) (*n* = 5 batches of cells). (c) Representative Western blot gel documents showing cleaved caspase-1 increased in HSCs treated with PA but blocked by FTZ. (d) Densitometric quantitation of immunoreactive bands showing a statistically significant increase in cleaved caspase-1 after PA-treated cells (*n* = 6 batches of cells). (e) ELISA of IL-1*β* levels from cell media showing a statistically significant increase induced by PA and decreased by FTZ (*n* = 5 batches of cells). ^∗^ *p* < 0.05 versus Vehl-Ctrl group; ^#^ *p* < 0.05 versus PA-Vehl group.

To explore the mechanisms of NLRP3 inflammasome formation and activation in HSCs, we used palmitic acid (PA), one of the major components of saturated fatty acid, to induce lipid deposition to examine the role of NLRP3 inflammasome activation and related NASH-like changes in these cells. Confocal microscopic analysis found that the colocalization of NLRP3 with caspase-1 or ASC increased in HSCs upon PA stimulation, which indicates the aggregation or assembly of these inflammasome molecules indeed occurs in response to PA stimulation. In HSCs pretreated with FTZ extracts, there was no colocalization of NLRP3 with caspase-1 or ASC in HSCs stimulated by PA (Figure 4(a)). The correlation coefficient of NLRP3 with ASC or caspase-1 showed that the colocalization of NLRP3 molecules increased significantly in HSCs stimulated by PA, which was completely blocked by FTZ, suggesting that FTZ is able to block NLRP3 inflammasome formation induced by PA in HSCs (Figure 4(b)).

We also determined NLRP3 inflammasome activation by analysis of active caspase-1 and IL-1*β* production in HSCs with and without pretreatment with FTZ. As shown in Figures 4(c) and 4(d), PA significantly increased the level of cleaved or active caspase-1 (15 kDa) and FTZ completely blocked this PA-induced increase in active caspase-1 level. Correspondingly, biochemical analysis showed that PA induced a significant increase in IL-1*β* production from HSCs, which was also completely blocked by FTZ treatment. The inhibition of PA-induced IL-1*β* production by FTZ was similar to the effects of NAC, an often used antioxidant for suppression of NLRP3 inflammasome activation (Figure 4(e)).

### 3.5. Effects of Mouse Nlrp3 Gene Silencing on PA-Induced NLRP3 Inflammasome Activation with and without FTZ

**Figure 5:**
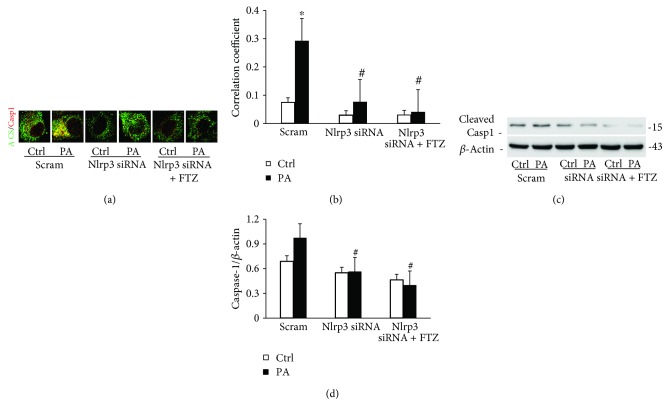
Nlrp3 gene silencing inhibited PA-induced NLRP3 inflammasomes formation and activation in HSCs with and without FTZ treatment. (a) Representative fluorescence confocal microscopic images showing the colocalization of ASC with caspase-1. (b) Correction coefficient (PCC) showing a statistically significant increase in colocalization of ASC with caspase-1 in PA-treated HSCs, which was suppressed by Nlrp3 siRNA and FTZ. (c) Representative Western blot gel documents showing cleaved caspase-1 increased in HSCs treated with PA but blocked by Nlrp3 siRNA and FTZ. (d) Densitometric quantitation of immunoreactive bands showing a statistically significant decrease in cleaved caspase-1 by using Nlrp3 siRNA and FTZ (*n* = 3 batches of cells). ^∗^ *p* < 0.05 versus Ctrl-scram group; ^#^ *p* < 0.05 versus PA-scram group.

**Figure 6:**
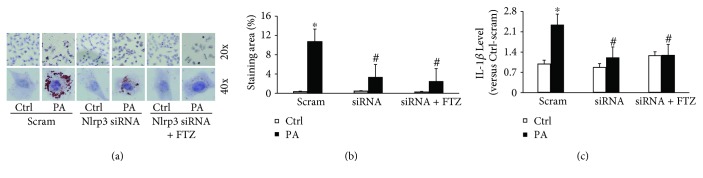
Nlrp3 gene silencing inhibited PA-induced NLRP3 inflammasome production and steatosis in HSCs with and without FTZ treatment. (a) Representative oil red O images showing positive staining of lipid deposition in PA-treated HSCs, which was suppressed by Nlrp3 siRNA and FTZ. (b) Staining area of lipid deposition showing a statistically significant increase induced by PA, which was reduced by Nlrp3 siRNA and FTZ. (c) ELISA of IL-1*β* levels from cell media showing a statistically significant decreased by Nlrp3 siRNA and with FTZ (*n* = 3 batches of cells). ^∗^ *p* < 0.05 versus Ctrl-scram group; ^#^ *p* < 0.05 versus PA-scram group.

Knockdown of mouse Nlrp3 mRNA level by Nlrp3 siRNA in HSCs remarkably inhibited PA-induced colocalization of ASC with caspase-1 (Figures 5(a) and 5(b)) and attenuated PA-increased level of cleaved caspase-1 (Figure 5(c)) and both blocked by FTZ. Consistent with these findings, Nlrp3 gene silencing with or without FTZ blocked IL-1*β* production in HSCs (Figures 6(a) and 6(b)) and PA-induced steatosis (Figure 6(c)). These results from NLRP3 gene silencing further support that it is the NLRP3 inflammasome that contributes to inflammatory response during NASH of this model and that the effective treatment of FTZ may be due to inhibition of the NLRP3 inflammasome activation.

### 3.6. Involvement of Redox Signaling in PA-Induced NLRP3 Inflammasome Activation in HSCs and the Effect of FTZ

**Figure 7:**
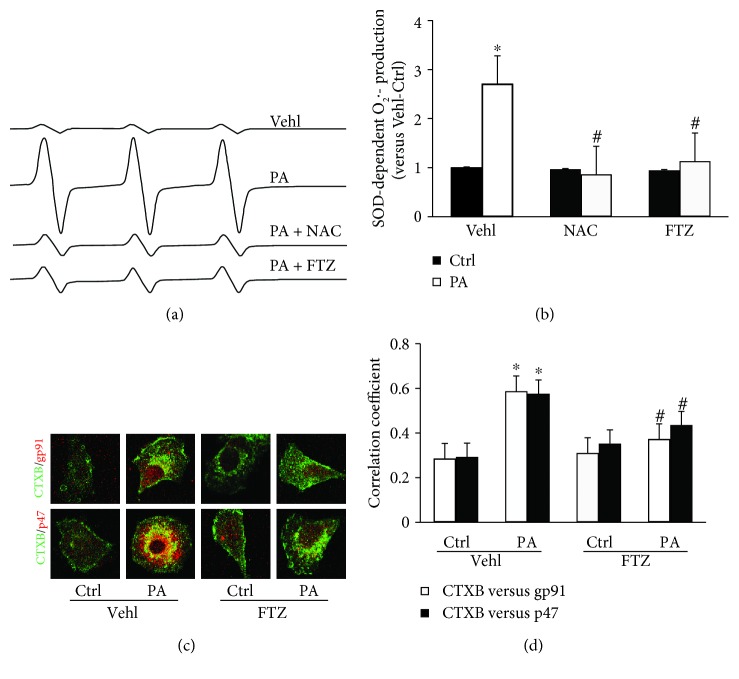
Superoxide production and NADPH oxidase-membrane raft (MR) clustering in HSCs induced by PA with and without FTZ. (a) Representative ESR traces of superoxide (O_2_^•−^) trapped by CMH using NADPH as substrate upon PA stimulation in HSCs. (b) The bar graph summarizing ESR data, showing that PA enhanced O_2_^•−^ production in HSCs, which was attenuated by FTZ and NAC (*n* = 5 batches of cells). (c) Representative fluorescence confocal microscopic images showing the colocalization of MR component labeled by CTXB with NADPH oxidase subunit gp91 or p47. (d) Correction coefficient (PCC) showing a statistically significant increase in colocalization of CTXB with gp91 or p47 in PA-treated HSCs, which was suppressed by FTZ or NAC treatment (*n* = 5 batches of cells). ^∗^ *p* < 0.05 versus Ctrl-Vehl group; ^#^ *p* < 0.05 versus PA-Vehl group.

Since previous studies showed that the MR redox signaling platforms and redox signaling are involved in inflammasome activation [24–26], we determined whether PA-induced NLRP3 inflammasome activation is associated with this redox regulatory pathway. As measured by ESR, O_2_^•−^ production significantly increased when HSCs were stimulated by PA, which was shown in largely enhanced reactive signals in ESR chromatography. FTZ had no effects on basal O_2_^•−^ production but inhibited PA-induced increases in ESR signal (Figure 7(a)). By calculation and nomination to SOD-sensitive components in ESR signals, PA-induced O_2_^•−^ production in HSCs was found to be completely blocked by FTZ. This FTZ-mediated inhibitory effects on PA-induced O_2_^•−^ production were similar to NAC (Figure 7(b)).

We next used Alexa Fluor 488-labeled CTXB (a MR marker) and anti-gp91 or anti-p47 antibody (NADPH oxidase (NOX) subunits) to measure clustering of MRs with both NOX subunits, which indicates MR redox signaling platform formation to produce O_2_^•−^. PA stimulation was found to significantly increase MR clustering with gp91 or p47 NOX subunits, as shown by the yellow patches on the HSC membrane (Figure 7(c)). It is well known that this MR redox platform formation led to the activation of NOX and subsequent generation of O_2_^•−^. These results were summarized in Figure 7(d), clearly showing that PA stimulated MR redox signaling platform formation in HSC membrane and FTZ blocked this PA effect.

### 3.7. Contribution of HMGB1 from NLRP3 Inflammasome Activation to PA-Induced Lipid Deposition and to the Beneficial Effects of FTZ

**Figure 8:**
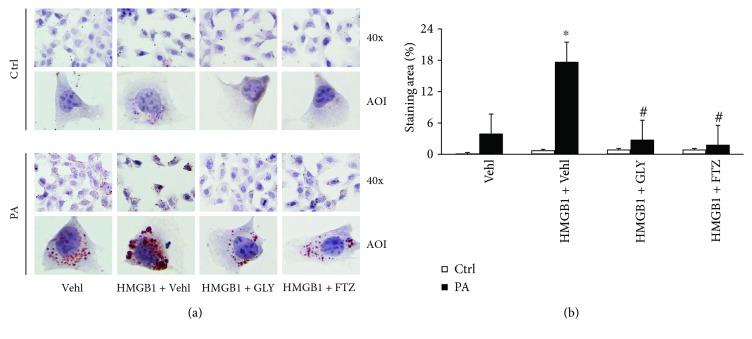
Involvement of HMGB1 in lipid deposition in HSCs treated with PA with and without FTZ treatment. (a) Representative oil red O images showing positive staining of lipid deposition in PA-treated HSCs, which was enhanced by HMGB1 but suppressed by FTZ and GLY, an inhibitor of HMGB1. (b) Staining area of lipid deposition showing a statistically significant increase induced by PA, which was enhanced by HMGB1, but reduced by FTZ and HMGB1 inhibitor, GLY (*n* = 5 batches of cells). ^∗^ *p* < 0.05 versus PA-Vehl group; ^#^ *p* < 0.05 versus PA-HMGB1-Vehl group.

As described above, our animal studies found that NLRP3 inflammasome activation not only participated in HFD-induced hepatitis and liver fibrosis but also caused steatosis in the liver. FTZ could block the development of both steatosis and sterile hepatitis in the liver. It seems that NLRP3 inflammasome activation also has uncanonical effects in NASH development which is beyond inflammation. To test this hypothesis, we addressed the role of HMGB1, an NLRP3 inflammasome product, which has been reported to increase lipid deposition [8]. As shown in Figure 8(a), the representative oil red O staining showed that PA resulted in strong staining of lipids in HSCs, which was blocked by FTZ pretreatment. The effects of PA can be mimicked by HMGB1 but inhibited by HMGB1 inhibitor, glycyrrhizin (GLY). The quantitative measurement of oil red O staining areas was summarized in Figure 8(b), showing that PA increased lipid deposition, which was blocked by FTZ. The PA-induced lipid deposition was significantly enhanced by the addition of HMGB1 but blocked by GLY, even in the presence of HMGB1.

## 4. Discussion

The current study provides several important findings. First, it demonstrated that NLRP3 formation and activation was enhanced during the development of NASH, which was confirmed in both *in vivo* animal and *in vitro* cell studies. Secondly, we verified the FTZ effect on NLRP3 inflammasome by using siRNA knocking down Nlrp3 gene. Thirdly, it was found that the NLRP3 inflammasome activation was inhibited by FTZ, which was accompanied by a reduction of liver lipid deposition and fibrogenic phenotype changed. It is indicated that FTZ exerts its beneficial action to not only prevent the inflammatory response but also suppress steatosis during HFD. Lastly, it showed that MR redox signaling platform formation and associated NADPH oxidase activation were involved in NLRP3 inflammasome activation and thereby contributed to the development of NASH. This MR redox signaling mechanism is responsible for liver inflammasome activation and NASH participated in the beneficial effects of FTZ against lipid deposition, inflammation, and fibrosis in the liver. The results suggest that NLRP3 inflammasome formation and activation via MR raft redox signaling platforms play a critical role in the initiation and progression of NASH and that FTZ exerts its beneficial action through inhibition of NLRP3 inflammasome activation in the liver.

We first determined whether NLRP3 inflammasome formation and activation occurred during the development of NASH using a mouse model of steatosis induced by HFD. NAFLD activity scores in the 8-week HFD group are among 3 to 4, which considered borderline or positive for NASH which also considered as the early stage of NASH. It was found that this inflammasome was formed and activated in the liver, which was companied by sterile inflammation and fibrogenesis, indicating the typical pathological changes of NASH. In isolated and cultured HSCs, we further showed that PA significantly enhanced NLRP3 inflammasome formation and activation, which were also with lipid deposition and fibrogenic phenotype changed in HSCs. These results tell us that NLRP3 inflammasome activation in the liver or in HSCs may be an important early pathogenic mechanism to turn on the inflammatory response and thereby instigate liver fibrosis during NASH. This is consistent with some previous reports indicating that NLRP3 inflammasome activation plays a fundamental role in the development of NASH [3]. In other liver fibrotic animal models such as alcoholic steatosis and cirrhosis [2], viral hepatitis-induced cirrhosis [27] and hepatic fibrosis during Schistosoma J infection [4], NLRP3 inflammasome activation has also been reported to either trigger or modulate hepatic inflammation leading to fibrosis [28]. The Sirius red staining in histological liver sections showed no significant liver fibrosis and cirrhosis after 8 weeks of HFD in this mouse model. However, some staining could be seen in sinus area with more HSCs. It is possible that some HSCs become fibrotic even at weeks of HFD when NLRP3 inflammasomes are activated (Supplementary Figure 3). Taken together, it is clear that NLRP3 inflammasome as intracellular inflammatory machinery is essential for the development of NASH and other liver fibrotic diseases.

Although chronic inflammation is a hallmark of NASH, the classic anti-inflammatory medicines, such as commonly used indole and arylpropionic acid derivatives are not very efficient in the prevention or treatment of NASH [29]. This may be mainly because these classical anti-inflammatory strategies may not target the noninflammatory or noncanonical effects during NLRP3 inflammasome activation in the development of NASH [6], and therefore they may only have a limited therapeutic effect. This led us to think that the NLRP3 inflammasome and its regulatory pathways may be an ideal target for treatment of chronic degenerative diseases like NASH with multiple pathological processes. Given our long-lasting interest in FTZ, a widely used herbal remedy for metabolic syndrome and hyperlipidemia and related complications in China, we tested whether FTZ exerts its action through inhibition of NLRP3 inflammasome formation and activation. In our studies with an animal model of NASH induced by HFD and with cultured HSCs stimulated by PA, we demonstrated that FTZ remarkably inhibited the formation and activation of NLRP3 inflammasomes. This inhibitory effects of FTZ on NLRP3 inflammasome activation reduced both hepatic inflammation and steatosis. Furthermore, this NLRP3 inhibition was also found to abrogate fibrotic process in the liver during NASH. It appears that FTZ indeed exerts its beneficial action in preventing NASH through suppression of NLRP3 inflammasome activation in the liver. To our knowledge, the findings from the present study for the first time link the therapeutic effect of FTZ to NLRP3 inflammasome activation, which serves as a molecular mechanism of the FTZ action.

FTZ has been prescribed over the last 15 years for treatment of hyperlipidemia and metabolic syndrome and related complications such as atherosclerosis and NASH [11, 12]. Previous studies have demonstrated that FTZ attenuated metabolic syndrome- (MS-) associated symptoms and pathological changes in tissues or cells, which was attributed to the decreases in the plasma levels of glucose and lipids [30]. In addition, some studies have shown that FTZ attenuated the downregulation of PI3K p85 mRNA and IRS1 protein in both insulin-resistant HepG2 cells and MS rats [30]. In a recent study, some components of FTZ were found to prevent the development of fatty liver in rats [13]. However, these studies did not elucidate the precise mechanisms how FTZ works to inhibit liver inflammation and to change fibrogenic phenotype. In the present study, we not only demonstrated that the suppression of NLRP3 inflammasome as an intracellular inflammatory machinery during NASH is an underlying mechanism responsible for the anti-inflammatory action of FTZ but also interestingly confirmed that FTZ inhibits lipid deposition in liver cells by the blockade of NLRP3 inflammasome-mediated HMGB1 production. It is believed that through inhibition of NLRP3 inflammasome activation FTZ may work to interfere with the early lipid deposition process and the late induction of hepatic inflammation and fibrosis during the progression of NASH. In previous studies, cholesterol, free fatty acids, and triglycerides were found to store in HSCs, which may activate these cells to become fibrogenic initiating or promoting liver fibrosis. Activated HSCs could sensitize the cell injury to further enhance lipid accumulation when there is increased intake of cholesterol, which may lead a vicious cycle in NASH, namely, accumulated lipids activating HSCs and the latter resulting in more accumulation of lipids in these cells [31]. The results from the present study provide the first experimental evidence that HMGB1 release derived from NLRP3 inflammasome-dependent caspase-1 activity may be involved in this lipid deposition process as shown in some other studies [5, 32, 33]. This action of HMGB1 can be blocked by FTZ treatment.

**Figure 9:**
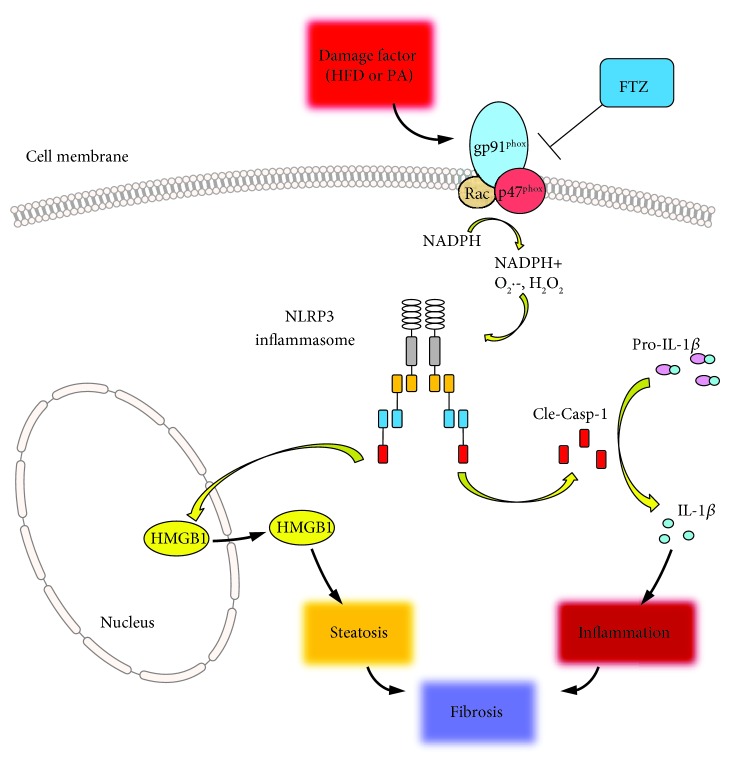
A schematic illustration of plausible mechanisms by which FTZ inhibits the NLRP3 inflammasome formation and activation and thus ameliorates steatohepatitis or NASH.

We also explored the mechanisms by which FTZ inhibits NLRP3 inflammasome formation and activation and thereby prevent NASH development. It was demonstrated that FTZ inhibited MR redox signaling platform formation to produce O_2_^•−^. This action further blocked NLRP3 inflammasome formation and activation in HFD-induced NASH in mice or in PA-stimulated HSCs. Although there are reports that in chronic liver disease oxidative stress has a clear role in HSC activation triggering fibrotic process [34], the results from the present study are the first to clarify that local oxidative stress may induce HSC activation and liver fibrosis through NLRP3 inflammasomes. FTZ may target this very early event of NASH to prevent the degenerative outcome of this liver disease. All these roles of NLRP3 inflammasomes in NASH and the beneficial effects of FTZ are diagrammatically summarized in Figure 9.

In summary, the present study demonstrated that increased NLRP3 inflammasome formation and activation are an important pathogenic mechanism initiating and promoting the development of NASH. FTZ suppressed this NLRP3 inflammasome activation to prevent steatosis, hepatic inflammation, and fibrogenic phenotype changed. This beneficial action of FTZ through the inhibition of NLRP3 inflammasome activation was not only associated with suppression of liver inflammation and HSCs activation leading to fibrogenesis but also with the early event of lipid deposition triggering steatosis. Furthermore, we showed that the inhibitory effect of FTZ on the NLRP3 inflammasome activation was due to blockade of MR redox signaling platform formation and subsequent O_2_^•−^ production.

## 5. Conclusion

The present study demonstrates that FTZ extracts inhibit NASH by its action on both inflammatory response and lipid metabolism associated with NLRP3 inflammasome activation in the liver. Targeting NLRP3 inflammasome to reduce steatosis, sterile liver inflammation, and consequent fibrosis may be an underlying mechanism for the therapeutic action of FTZ on NASH and possibly on other end-organ damage induced by metabolic syndrome.

## Data Availability

The data used to support the findings of this study are available from the corresponding author upon request.

## Supplementary Materials

Supplementary Materials Inflammasome activation is the important role in the early pathogenic mechanism of inflammation. We focused on the mechanism of NLRP3 inflammasome which is an intracellular inflammatory machinery in the early stage of NASH. The detail information of NAS was shown in Supplementary Figure 1(b). It showed that NAS had statistically significant increased after HFD for 8 weeks. NAS in the HFD group is among 3 to 4, which considered borderline or positive for NASH which also considered as the early stage of NASH. The result about body weight of HFD mice was increased compared to ND mice but none increased after FTZ treatment (Supplementary Figure 1(a)). The results of AST activity assay and blood glucose were not statistically different among each group, suggesting that there was no abnormal liver function and metabolic syndrome in this HFD-induced early NASH model (Supplementary Figure 2). In the same time, there was no significant liver fibrosis and cirrhosis after 8 weeks of HFD in this mouse model by Sirius red staining (Supplementary Figure 3).
