# Supplementary material for: NLRP3 Inflammasome Formation and Activation in Nonalcoholic Steatohepatitis: Therapeutic Target for Antimetabolic Syndrome Remedy FTZ
Source: Oxid Med Cell Longev. 2018 Jul 22;2018:2901871. doi: 10.1155/2018/2901871 (PMC6081604; doi:10.1155/2018/2901871)
Supplement: Supplementary Materials — Inflammasome activation is the important role in the early pathogenic mechanism of inflammation. We focused on the mechanism of NLRP3 inflammasome which is an intracellular inflammatory machinery in the early stage of NASH. The detail information of NAS was shown in Supplementary Figure 1(b). It showed that NAS had statistically significant increased after HFD for 8 weeks. NAS in the HFD group is among 3 to 4, which considered borderline or positive for NASH which also considered as the early stage of NASH. The result about body weight of HFD mice was increased compared to ND mice but none increased after FTZ treatment (Supplementary Figure 1(a)). The results of AST activity assay and blood glucose were not statistically different among each group, suggesting that there was no abnormal liver function and metabolic syndrome in this HFD-induced early NASH model (Supplementary Figure 2). In the same time, there was no significant liver fibrosis and cirrhosis after 8 weeks of HFD in this mouse model by Sirius red staining (Supplementary Figure 3). [file 2901871.f1.docx]

**Supplementary Figure 1.** The body weight and detail of NAS of mice in HFD with and without FTZ.

(a) Body weight of mice showing statistically significant increase in HFD groups, but decrease after FTZ by gavage (the yellow arrow is the time started to use FTZ). (b) NAFLD activity score represents the sum of scores for steatosis, lobular inflammation, and ballooning, and ranges from 0-8. NAFLD activity score in HFD group is among 3 to 4, which considered borderline or positive for NASH. But the score decrease to less than 3 after FTZ treatment, especially in the scores of steatosis and lobular inflammation of mice (n=4 mice per group).

**Supplementary Figure 2.** The blood glucose and AST activity assay of mice in HFD with and without FTZ.

(a) Blood glucose of mice showing no statistically significant in different group. (b) ELISA of AST activity assay of mice showing no statistically significant different group (n=4 mice per group).

**Supplementary Figure 3.** Sirius red staining in the liver from mice on the HFD with and without FTZ treatment.

(a) Representative sirius red staining images showing no fibrosis in mice with 8 weeks HFD, but it seems that staining enhanced in HFD group but less with FTZ treatment. (b) Summarized data depicting no statistically significant among each group (n=4 mice per group).
